# Case Report: Methimazole-Induced Parotitis - An Unusual Presentation

**DOI:** 10.12688/f1000research.149569.2

**Published:** 2024-08-27

**Authors:** Ricky Rana, Emily Krier, Abubakar Tauseef, Jalal Dufani

**Affiliations:** 1 Internal Medicine, CHI Health Creighton University Medical Center Bergan Mercy, Omaha, Nebraska, 68106, USA

**Keywords:** methimazole, drug-induced reaction, parotitis, hyperthyroidsm

## Abstract

A 56-year-old female with a medical history of unspecified hyperthyroidism and a recent thyroid storm presented to the ED with tachycardia, hypertension, and bilateral enlarged parotid glands. During a previous hospitalization, she was diagnosed with unspecified hyperthyroidism and started on methimazole. During hospitalization, laboratory findings suggested Graves’ disease with an acute thyroid storm. The patient also complained of enlarged parotid glands bilaterally. CT tomography of the neck revealed no calculi of the parotid glands but showed extensive fatty replacement, possibly related to methimazole use. Treatment with propranolol and IV hydrocortisone improved thyroid function. Due to the suspicion of methimazole-induced parotitis, she was transitioned to a reduced methimazole dosage for treatment of Graves’ disease, which subsequently improved her parotitis.

Methimazole, the standard initial treatment for Graves’ disease, is generally well-tolerated. It can cause adverse reactions; however, parotitis is very rare and has been documented in only a few case reports. Owing to the limited number of reports, its incidence is currently unknown. Here, we present a case of methimazole-induced parotitis as an unusual presentation of thyroid storm.

Drug-induced reactions can only be considered once common causes of parotitis such as viral infection, obstruction, and autoimmune diseases are ruled out. Treatment involves dosage adjustments and supportive care. Methimazole-induced parotitis is often misdiagnosed and overlooked because of the lack of reported cases. This necessitates future research into the reaction mechanisms and optimal treatment.

## Introduction

Parotitis, or inflammation of the parotid glands, is one of the most common disorders of the salivary glands and is generally caused by viral infections, obstruction of calculi, autoimmune diseases, and adverse effects of medications. The medications most associated with parotid gland enlargement include clozapine and phenylbutazone. ^1^^–^^3^ Other drugs such as methimazole have been implicated in the development of parotitis; however, methimazole-induced acute parotitis is rare and easily misdiagnosed. Therefore, the incidence of methimazole-induced parotitis remains unknown. Based on limited experience, it generally resolves once the methimazole is stopped or the dose is decreased. Here, we present the case of a 56-year-old female who presented to the Emergency Department with thyroid storm and an incidental finding of methimazole-induced parotitis.

## Case presentation

A 56-year-old female with a medical history of hypertension, gastroesophageal reflux disease, type 2 diabetes, unspecified hyperthyroidism, and recent thyroid storm was transferred from an outside hospital emergency department for the management of thyroid storm due to medication non-compliance. She had been hospitalized a few months prior for epigastric discomfort, nausea, tachycardia with a heart rate of 140 beats/min, and hypertension with a blood pressure of 175/80 mmHg. During that hospitalization, she was diagnosed with thyroid storm and was started on methimazole 10 mg twice a day and atenolol 50 mg daily which she took at home for 5 months. Patient was subsequently lost to follow up with endocrinology.

During this hospitalization, the patient presented with recurrent symptoms of thyroid storm. Physical examination was unremarkable, except for a flushed and ill-appearing, disoriented female with bilateral, non-tender, enlarged parotid glands, and no sign of exophthalmos. A repeat laboratory investigation showed mild transaminitis, TSH level of 0.008 IU/ml with T3 of 594 ng/dl, free T4 of 6.79 ng/dl, thyroid peroxidase antibody over 1300 u/ml and an elevated TSH receptor antibody of 9.86 IU/L. The rest of the autoimmune and viral workup, including mumps serology, was unremarkable. The patient’s history, physical examination, and laboratory findings were consistent with Graves’ disease presenting with acute thyroid storm secondary to medication noncompliance. The patient presented with bilateral swelling at the level of the jaw. Ultrasound and CT scan of the neck showed no obstructing calculi in the parotid glands but showed extensive fatty replacement of both parotid glands with parotitis.

For treatment of parotitis, the patient underwent conservative measures including warm compression, analgesia, and increased fluid intake. For the treatment of thyroid storm, she was started on propranolol 40 mg twice daily and methimazole 20 mg three times daily for 12 days. Her dose of propranolol was briefly increased to 60 mg every 6 hours for 2 days and 80 mg every 6 hours for 3 days before weaning back down. She was later transitioned to IV hydrocortisone 100 mg every eight hours and propylthiouracil 200 mg every 4 h per endocrinologist recommendation. Thyroid function gradually improved over 18 days of this treatment regimen. By hospital week 4, the patient was stable on 40 mg propranolol twice daily, propylthiouracil 100 mg twice daily, and maintained her dose of IV hydrocortisone 100 mg every eight hours for 7 days until her thyroid was surgically removed to prevent further complications. During her hospitalization, the patient was transitioned to propylthiouracil therapy for the management of Graves’ disease due to a high suspicion of methimazole-induced parotitis rather than parotitis induced by the thyroid storm itself. Her parotitis resolved with conservative treatment and termination of methimazole, the likely causative agent.

## Case discussion

Methimazole is generally a well-tolerated treatment for Graves’ disease (GD). Among the patients who develop adverse drug reactions, 75% occur during the first 6 months of treatment. ^4^ Common adverse effects include skin reactions, gastrointestinal upset, and agranulocytosis; however, they rarely manifest as pancreatitis or parotitis. ^3^^,^^5^ While pancreatitis is a well-documented adverse effect of the drug, occurring with a risk ranging between 0.02% and 0.56%, parotitis is a much rarer side effect, with few reported cases. ^6^ Based on a review of the literature and using the modified Naranjo probability scale for drug-induced parotitis, medications that met the criteria for establishing causality included l-asparaginase, clozapine, and phenylbutazone. ^1^ Owing to the lack of reported cases of methimazole-induced parotitis, there is insufficient evidence to determine causality. In addition, owing to the paucity of reported cases, the incidence of methimazole-induced parotitis is currently unknown, and uncertainty surrounds the precise mechanism that drives this adverse reaction. There are several common causes of parotitis including duct obstruction, infectious organisms, inflammatory conditions, neoplasm and medication. ^7^ Drug-induced hypersensitivity reactions, inflammation, and altered salivary gland function are some of the proposed hypotheses for medication induced parotitis. ^2^^,^^8^ With the symptoms appearing days or weeks after starting methimazole therapy, it seems more likely to be due to an idiosyncratic reaction rather than a dose-dependent reaction. It has been hypothesized that certain peptides are modified by methimazole and act as antigens that are then presented by specific HLA molecules in susceptible patients to trigger a cellular or humoral immune response. ^1^^,^^2^ This explanation for an immune-mediated response is further strengthened by the fact that both pancreatitis and parotitis are known side effects. It has been shown that both glands share many histological and functional similarities. ^9^ Furthermore, it is thought that a shared pancreatic and parotid peptide is involved in the development of a hypersensitivity reaction that can injure these glands.

We conducted a literature review to find other cases of methimazole induced parotitis ( Table 1). JAMA and PubMed were searched using the terms parotitis and methimazole. Two studies were found describing a similar clinical vignette to this patient.

**Table 1.** 

Author	Case Title	Year	Description	Outcome
Moehlig RC ^8^	Parotid Gland Swelling due to Methimazole	1969	62-year old woman with bilateral parotitis following initiation of methimazole for hyperthyroidism	Swelling disappeared within 3 days of discontinuing methimazole
Taguchi M, Yokota M, Koyano H, et al. ^5^	Acute pancreatitis and parotitis induced by methimazole in a patient with graves’ disease	2001	66-year-old woman with bilateral parotitis 3 weeks after initiation of methimazole for thyrotoxicosis	Methimazole was discontinued and swelling disappeared over the next week
Rana R, Krier EN, Tauseef A, Dufani J	Methimazole-Induced Parotitis - An Unusual Presentation	2024	56-year-old female with bilateral parotitis after initiating methimazole for treatment of thyroid storm	Methimazole was discontinued and supportive treatment was initiated. Swelling disappeared over the next few weeks

In patients with parotitis, common causes such as calculi, viral infection, autoimmune diseases, and neoplastic processes should be ruled out before diagnosing medication-induced parotitis. Serological testing, including polymerase chain reaction, cytology, and imaging, is required to rule out these conditions.

Severe hyperthyroidism and thyrotoxicosis can also cause parotitis. Studies have shown that patients with hyperthyroidism show reduced salivary production and about half of the patients studied had histopathologic and clinical signs of parotitis. ^10^ Thyrotoxicosis can also result in other clinical and laboratory abnormalities such as elevated liver blood tests, neutropenia, and thymic hyperplasia. ^11^^,^^12^

Methimazole-induced parotid gland inflammation can be managed by reducing the dosing of the offending drug, as observed in this case. Similar adverse reactions can be prevented by initially starting patients on a low dose of methimazole and gradually increasing the dosage over a few weeks to prevent adverse effects. If parotitis develops, the symptoms can be alleviated with supportive care, including fluids, warm compresses, and analgesics. If the patient does not respond to treatment, it is recommended to rule out other causes of parotid enlargement, including abscesses or bacterial infections. Methimazole is an efficacious treatment with a long-term remission rate for Graves’ disease after treatment, ranging between 30% and 70%. ^13^

## Conclusion

Methimazole is the standard initial treatment for Graves’ disease and is generally well-tolerated in a wide variety of patients. It can cause some adverse reactions, most commonly skin rashes, and gastrointestinal upset; however, parotitis is a rarely reported adverse reaction documented in only a handful of case reports upon a review of the literature.

Once the common causes of parotid gland inflammation, such as viral infection, obstruction of calculi, and autoimmune disease, are ruled out, a drug-induced reaction can be considered. Treatment generally consists of stopping or decreasing the dose of the offending agent as well as supportive care such as fluids, warm compresses, and analgesics.

Owing to the lack of reported cases, methimazole-induced acute parotitis is easily misdiagnosed and can be overlooked. Therefore, further research is needed to determine the precise mechanism causing this adverse drug reaction as well as the optimal treatment to prevent lasting damage.

## Consent

Written informed consent for publication of their clinical details was obtained from the patient.

## Data Availability

No data is associated with this article.
