# Supplementary material for: Case Report: Methimazole-Induced Parotitis - An Unusual Presentation
Source: F1000Res. 2025 Apr 4;13:447. Originally published 2024 May 3. [Version 3] doi: 10.12688/f1000research.149569.3 (PMC12032518; doi:10.12688/f1000research.149569.3)
Supplement: Peer review history

# Reviewer report: Reviewer response for version 3

**DOI:** 10.5256/f1000research.179678.r375575

**Published:** 2025-04-26

**Authors:** Sumayah Faruq Kasim

**Affiliations:** 1 Middle Technical University, Baghdad, Iraq

While the manuscript presents a compelling and rare clinical scenario, the overall impact of the case would be significantly strengthened by the inclusion of supporting clinical imaging, a more detailed immunological evaluation, and a more comprehensive exclusion of alternative diagnoses. These additions would not only enhance diagnostic clarity but also reinforce the case's relevance to broader clinical practice.

 For future case reports, we kindly suggest considering the early integration of:

- **Imaging documentation** (e.g., ultrasound, CT scans, or photographic evidence) to visually support physical findings.
- **Immunological panels** (e.g., ANA, anti-Ro/SSA, anti-La/SSB, IgG4) when autoimmune or idiopathic causes are in the differential.
- **A standardized diagnostic checklist** to systematically rule out infectious, autoimmune, obstructive, and neoplastic etiologies of parotitis.

Incorporating these elements prospectively, at the time of patient evaluation and documentation, will enhance the rigor and reproducibility of the report, while improving its educational value for future readers.

 Actually, the authors made good-faith efforts to respond, especially in discussion depth and literature comparison. I recommended acceptance of the article.

Are enough details provided of any physical examination and diagnostic tests, treatment given and outcomes?

Yes

Is the case presented with sufficient detail to be useful for other practitioners?

Yes

Is sufficient discussion included of the importance of the findings and their relevance to future understanding of disease processes, diagnosis or treatment?

Yes

Is the background of the case’s history and progression described in sufficient detail?

Yes

Reviewer Expertise:

Physiology of Endocrine and Reproductive System

I confirm that I have read this submission and believe that I have an appropriate level of expertise to confirm that it is of an acceptable scientific standard.

# Reviewer report: Reviewer response for version 3

**DOI:** 10.5256/f1000research.179678.r375574

**Published:** 2025-04-25

**Authors:** Jeyakantha Ratnasingam

**Affiliations:** 1 University Malaya, Kuala Lumpur, Malaysia

The article is more complete now in terms of history, chronology of events and progress.

 However, the authors still need to acknowledge that the cause of parotitis in this case could have been due to the disease itself rather than methimazole. .

 Also important to highlight if propylthiouracil could also cause similar parotitis, as this has been reported as well. In such instance how would you treat this case ?

Are enough details provided of any physical examination and diagnostic tests, treatment given and outcomes?

No

Is the case presented with sufficient detail to be useful for other practitioners?

No

Is sufficient discussion included of the importance of the findings and their relevance to future understanding of disease processes, diagnosis or treatment?

No

Is the background of the case’s history and progression described in sufficient detail?

No

Reviewer Expertise:

Endocrinology, Osteoporosis, Obesity and Diabetes

I confirm that I have read this submission and believe that I have an appropriate level of expertise to confirm that it is of an acceptable scientific standard, however I have significant reservations, as outlined above.

# Reviewer report: Reviewer response for version 2

**DOI:** 10.5256/f1000research.170633.r367921

**Published:** 2025-03-19

**Authors:** Jeyakantha Ratnasingam

**Affiliations:** 1 University Malaya, Kuala Lumpur, Malaysia

The case attempts to highlight parotitis as an extremely rare side effect of methimazole, which will add to the current literature on this.

 However the case lacks clarity and the history does not point towards this. In fact the history is contradictory. It is stated that the patient had presented with thyroid storm and parotitis due to non-compliance to methimazole. As such, how do the authors conclude that the parotitis was due to methimazole and not from other causes ?

 No attempt was made to describe via images, or what other tests were done to rule out other more important and common causes of parotitis. 

 No specific testing was carried to exclude other causes of parotitis. 

 Authors have called the case as "unspecified" hyperthyroidism, despite clearly reporting elevated TSHRAb, which confirms Graves's disease. 

 The learning points were not clear. There was no clear message and recommendation on how to manage this in a real clinical scenario.

Are enough details provided of any physical examination and diagnostic tests, treatment given and outcomes?

No

Is the case presented with sufficient detail to be useful for other practitioners?

No

Is sufficient discussion included of the importance of the findings and their relevance to future understanding of disease processes, diagnosis or treatment?

No

Is the background of the case’s history and progression described in sufficient detail?

No

Reviewer Expertise:

Endocrinology, Osteoporosis, Obesity and Diabetes

I confirm that I have read this submission and believe that I have an appropriate level of expertise to state that I do not consider it to be of an acceptable scientific standard, for reasons outlined above.

# Reviewer report: Reviewer response for version 2

**DOI:** 10.5256/f1000research.170633.r360349

**Published:** 2025-02-07

**Authors:** Türkan Paşalı Kilit

**Affiliations:** 1 Kutahya Health Sciences University Faculty of Medicine, Kutahya, Turkey

We know that salivary gland involvement occurs in autoimmune thyroid diseases. Before diagnosing methimazole-related parotitis, was the patient subjected to parotid and thyroid gland scintigraphy?

 In patients with autoimmune thyroiditis Tc-99m MIBI uptake in salivary glands It has been shown that there is more involvement than normal individuals affected by autoimmunity.

 This situation was shown in the study named ‘’ Detection of the involvement of salivary glands in autoimmune diseases of thyroid gland by using tc-99m MIBI’’ (J Contemp Med 2017; 7(1):67-73 ORIGINAL ARTICLE DOI: 10.16899/gopctd.286073 Söylemez D [2017(Ref-1)] ).

 It is not clear whether it was after thyroid storm or after methimazole. It is known that severe thyrotoxicosis can cause parotitis. This issue needs to be clarified.

Are enough details provided of any physical examination and diagnostic tests, treatment given and outcomes?

Partly

Is the case presented with sufficient detail to be useful for other practitioners?

Partly

Is sufficient discussion included of the importance of the findings and their relevance to future understanding of disease processes, diagnosis or treatment?

Partly

Is the background of the case’s history and progression described in sufficient detail?

Yes

Reviewer Expertise:

diyabet mellitus Tiroid

I confirm that I have read this submission and believe that I have an appropriate level of expertise to state that I do not consider it to be of an acceptable scientific standard, for reasons outlined above.

## References

[1] : Otoimmün Tiroid Bezi Hastalıklarında Tükürük Bezi Tutulumunun Tc-99m MIBI İle Değerlendirilmesi. *Journal of Contemporary Medicine* .2017;7: 10.16899/gopctd.286073 10.16899/gopctd.286073

# Reviewer report: Reviewer response for version 2

**DOI:** 10.5256/f1000research.170633.r360342

**Published:** 2025-01-23

**Authors:** Sumayah Faruq Kasim

**Affiliations:** 1 Middle Technical University, Baghdad, Iraq

The manuscript effectively highlights a rare case of methimazole-induced parotitis, but it lacks an in-depth exploration of the immune-mediated or molecular mechanisms potentially underlying this reaction, such as HLA associations or inflammatory pathways. The discussion would benefit from a clearer differentiation between drug-induced parotitis and other potential causes like viral or autoimmune parotitis, with a stronger focus on diagnostic exclusions. Additionally, the absence of clinical photographs or imaging to visually support the case limits its impact. A more detailed comparison with previously reported cases, specifically elaborating on how this case contributes uniquely to the literature, is also needed. Lastly, the manuscript could be strengthened by expanding the conclusion with actionable recommendations for clinicians, such as early recognition strategies, alternative treatment approaches, and patient monitoring for rare adverse effects.

Are enough details provided of any physical examination and diagnostic tests, treatment given and outcomes?

Yes

Is the case presented with sufficient detail to be useful for other practitioners?

Yes

Is sufficient discussion included of the importance of the findings and their relevance to future understanding of disease processes, diagnosis or treatment?

Yes

Is the background of the case’s history and progression described in sufficient detail?

Yes

Reviewer Expertise:

Physiology of Endocrine and Reproductive System

I confirm that I have read this submission and believe that I have an appropriate level of expertise to confirm that it is of an acceptable scientific standard, however I have significant reservations, as outlined above.

# Response

**Published:** 2025-03-19

**Authors:** Ricky Rana

**Affiliations:** Internal Medicine, CHI Health Creighton University Medical Center Bergan Mercy, Omaha, Nebraska, USA

Thank you for this valuable feedback! We have taken into account your comments and included a more extensive diagnostic workup to exclude other causes and treatment plan in the conclusion section of the paper.  We have also added recommendations for patient monitoring and early diagnosis of adverse reactions such as this.

# Reviewer report: Reviewer response for version 2

**DOI:** 10.5256/f1000research.170633.r317450

**Published:** 2024-09-16

**Authors:** Lorenzo Scappaticcio

**Affiliations:** 1 AOU University of Campania, Naples, Italy

no further comments.

Are enough details provided of any physical examination and diagnostic tests, treatment given and outcomes?

No

Is the case presented with sufficient detail to be useful for other practitioners?

No

Is sufficient discussion included of the importance of the findings and their relevance to future understanding of disease processes, diagnosis or treatment?

No

Is the background of the case’s history and progression described in sufficient detail?

No

Reviewer Expertise:

NA

I confirm that I have read this submission and believe that I have an appropriate level of expertise to confirm that it is of an acceptable scientific standard.

# Reviewer report: Reviewer response for version 1

**DOI:** 10.5256/f1000research.164048.r304171

**Published:** 2024-08-14

**Authors:** Lorenzo Scappaticcio

**Affiliations:** 1 AOU University of Campania, Naples, Italy

The case is interesting, but not well-written.

 It needs the following things:

 - images (clinical and/or) of the enlarged parotid glands, showing how they decrease again.

 - the authors can not say which is the best mechanism under the enlargement of the parotids. Thus, they have to say in the discussion which are the main mechanisms, regardless of this case.

 - this case it can not be an example of idiosyncratic reaction, since the parotitis apparently disappeared after lowering the dose.

 -change the Title (since it is not clear if this happened after thyroid storm or methimazole).

 -in the discussion add some details and references regarding the possibility that severe thyrotoxicosis can induce parotitis.;

 -since you found only a found case reports, report: - where you found these cases (search strategy and online databases); -references of these case; - Table where you summaries the main findings of each case.

 -Table where you summarize the main findings of your case and your work.

 -cite the two relevant papers;

 (Scappaticcio et al.,2021)(Ref-1) (Scappaticcio et al.,2024)(Ref-2)

Are enough details provided of any physical examination and diagnostic tests, treatment given and outcomes?

No

Is the case presented with sufficient detail to be useful for other practitioners?

No

Is sufficient discussion included of the importance of the findings and their relevance to future understanding of disease processes, diagnosis or treatment?

No

Is the background of the case’s history and progression described in sufficient detail?

No

Reviewer Expertise:

thyrotoxicosis and thyroid nodules and cancers.

I confirm that I have read this submission and believe that I have an appropriate level of expertise to confirm that it is of an acceptable scientific standard, however I have significant reservations, as outlined above.

## References

[1] : Abnormal Liver Blood Tests in Patients with Hyperthyroidism: Systematic Review and Meta-Analysis. *Thyroid* .2021;31(6) : 10.1089/thy.2020.0715 884-894 10.1089/thy.2020.0715 33327837

[2] : Thymic hyperplasia is accurate to detect new-onset Graves' hyperthyroidism and resolves after restoring euthyroidism. *J Endocrinol Invest* .2024; 10.1007/s40618-024-02355-w 10.1007/s40618-024-02355-w 38553585 PMC11393051

# Response

**Published:** 2024-08-20

**Authors:** Ricky Rana

**Affiliations:** Internal Medicine, CHI Health Creighton University Medical Center Bergan Mercy, Omaha, Nebraska, USA

Thank you for your valuable feedback and comments. I have tried to address the points you brought up in the updated version of the article.
